# A nomogram for determining the disease-specific survival in Ewing sarcoma: a population study

**DOI:** 10.1186/s12885-019-5893-9

**Published:** 2019-07-05

**Authors:** Jun Zhang, Zhenyu Pan, Jin Yang, Xiaoni Yan, Yuanjie Li, Jun Lyu

**Affiliations:** 1 Clinical Research Center, The First Affiliated Hospital of Xi’an Jiaotong University, Xi’an, Shaanxi China; 2 School of Public Health, Xi’an Jiaotong University Health Science Center, Xi’an, Shaanxi China; 3 Department of Orthopaedics, Baoji Municipal Central Hospital, Baoji, Shaanxi China; 4 Department of Pharmacy, The Affiliated Children Hospital of Xi’an Jiaotong University, Xi’an, Shaanxi China; 5 Department of Gastroenterology, The First Affiliated Hospital of Xi’an Jiaotong University, Xi’an, Shaanxi China; 6 Department of Human Anatomy, Histology and Embryology, School of Basic Medical Sciences, Xi’an Jiaotosng University Health Science Center, Xi’an, Shaanxi China

**Keywords:** Nomogram, SEER, Disease-specific survival rate, Ewing sarcoma

## Abstract

**Background:**

We aimed to develop and validate a nomogram for predicting the disease-specific survival of Ewing sarcoma (ES) patients.

**Methods:**

The Surveillance, Epidemiology, and End Results (SEER) program database was used to identify ES from 1990 to 2015, in which the data was extracted from 18 registries in the US. Multivariate analysis performed using Cox proportional hazards regression models was performed on the training set to identify independent prognostic factors and construct a nomogram for the prediction of the 3-, 5-, and 10-year survival rates of patients with ES. The predictive values were compared by using concordance indexes (C-indexes), calibration plots, integrated discrimination improvement (IDI), net reclassification improvement (NRI), and decision curve analysis (DCA).

**Results:**

A total of 2,643 patients were identified. After multivariate Cox regression, a nomogram was established based on a new model containing the predictive variables of age, race, extent of disease, tumor size, and therapy of surgery. The new model provided better C-indexes (0.684 and 0.704 in the training and validation cohorts, respectively) than the model without therapy of surgery (0.661 and 0.668 in the training and validation cohorts, respectively). The good discrimination and calibration of the nomogram were demonstrated for both the training and validation cohorts. NRI and IDI were also improved. Finally, DCA demonstrated that the nomogram was clinically useful.

**Conclusion:**

We developed a reliable nomogram for determining the prognosis and treatment outcomes of patients with ES in the US. However, the proposed nomogram still requires external data verification in future applications, especially for regions outside the US.

## Background

Ewing sarcoma (ES) is the second most common malignant primary osseous sarcoma in children and adolescents [1]. Bone ES constitutes a family of malignant small round blue cell tumors with neuroectodermal origins, among which 85–90% have the classic t (11; 22) *EWS/FLI1* translocation [1, 2]. The overall survival (OS) rate for ES has improved remarkably over the past two decades due to advances in multimodality therapies. In the US, the 5-year survival rates increased from 16% in the 1970s to 39% in the 1990s/early 2000s among patients with metastatic disease. The survival parameter in patients with localized disease increased from 44 to 68% [3]. Despite these improvements, a large proportion of patients with ES still suffer from disease- or treatment-related morbidity or mortality. The early identification of high-risk patients can help provide adjuvant therapies or trial options. Given the clinical uniqueness of ES, prognostic tools are urgently needed to predict survival in ES patients accurately.

Nomograms are reliable and convenient tools for estimating tumor prognosis [4, 5]. In this study, we aimed to establish a comprehensive prognostic evaluation system. The data of ES patients in the Surveillance, Epidemiology, and End Results (SEER) program database registries during 1990–2015 were screened and extracted. We then analyzed the extracted data and subsequently created and validated a nomogram containing significant and reliable variables for quantifying the survival of ES patients.

## Methods

### Data source and inclusion criteria

We queried the SEER program database for ES records from 1990 to 2015 that covers approximately 30% of the US population and includes cases from 18 population-based registries [4]. Utilizing data from the SEER program does not require informed patient consent, and no case-identifying information is provided by the SEER cancer registries.

We searched for patients with ES by using the histological subtype code of “Ewing sarcoma” (9260/3) in the third edition of the International Classification of Diseases for Oncology. The patient demographic variables of interest included age at diagnosis (categorized into ≤30 years old and > 30 years old), sex, race, and marital status (categorized into married, single/domestic partner, or divorced/separated/widowed). A composite socioeconomic status (SES) score corresponding to the percentage of persons in the country living below the national poverty threshold in the official 2000 census [6] was divided into three levels by using previously reported cutoff points [6, 7], namely, < 10% (low poverty), 10–19.99% (moderate poverty), and ≥ 20% (high poverty). The year of diagnosis (YOD) was categorized into 1990s, 2000s and 2010s. EOD was categorized into confined, local invasion, metastasis, and unknown [8]. The primary site of ES was classified into extremity, axial skeleton, and others. Tumor size was grouped into ≤50 mm (small), > 50 and ≤ 100 mm (intermediate), and > 100 mm (large) [9]. Surgery, radiotherapy, and chemotherapy were categorized into received and not received/unknown. Patients with missing or unknown of survival period were excluded.

### Statistical analysis and nomogram construction

The categorical variables are expressed as frequencies and proportions and compared with the chi-square and Fisher’s exact tests. Multivariate analysis was performed by using Cox proportional hazards regression models to determine the factors associated with survival. On the basis of the predictive model with identified prognostic factors, a nomogram was constructed for predicting the 3-, 5-, and 10-year survival rates of ES patients.

### Nomogram validation and performance evaluation

The nomogram was validated by measuring the discrimination and calibration curves both internally (training cohort) and externally (validation cohort). Receiver operating characteristic (ROC) curves were generated to evaluate the performance of the nomogram on the basis of the areas under the ROC curves. The agreement between the predicted probability and actual outcome was evaluated via calibration plotting. The nomogram was subjected to bootstrapping validation (1,000 bootstrap resamples) to calculate a relatively corrected concordance index (C-index). The improvement in the predictive accuracy of the models with and without prognostic therapies was estimated by calculating the relative integrated discrimination improvement (IDI) and the net reclassification improvement (NRI), as described by Cook [10]. Finally, we evaluated the clinical usefulness and net benefit of the new predictive models by using decision curve analysis (DCA), as described by Vickers and Elkin [11].

Statistical analysis was conducted with SPSS (version 24.0; Chicago, IL, USA) and R (version 3.0.1; https://www.r-project.org/) softwares. *P* values < 0.05 of the two-sided tests were considered statistically significant.

## Results

### Demographic baseline characteristics

The application of the inclusion and exclusion criteria listed in the Materials and Methods resulted in the identification of 2,643 patients with ES in the SEER program database. The survival period was known for all of the included patients. For nomogram construction and validation, we randomly assigned 70 and 30% of the patients to the training (*n* = 1,850) and validation (*n* = 793) cohorts, respectively. The majority of patients were ≤ 30 years old (78.4 and 79.8% in the training and validation cohorts, respectively) and male (58.9 and 62.7%), white (88.1 and 88.8%), had a marital status of single/domestic partner (76.3 and 79.8%), had been diagnosed in the 2000s or 2010s (82.4 and 82.1%), had unknown EOD (67.1 and 67.0%), and had undergone surgery (60.9 and 59.9%) and chemotherapy (91.0 and 93.9%). The clinicopathological characteristics of all patients are listed in Table 1.

**Table 1:** Socio-demographic and clinical characteristics of patients in the study

Variable n (%)	Training Cohort	Validation Cohort
	(*n* = 1850)	(*n* = 793)
Age		
≤ 30 years	1451 (78.4)	633 (79.8)
>30 years	399 (21.6)	160 (20.2)
Sex		
Male	1089 (58.9)	497 (62.7)
Female	761 (41.1)	296 (37.3)
Race		
White	1630 (88.1)	704 (88.8)
Black	65 (3.5)	34 (4.3)
Other	155 (8.4)	55 (6.9)
Marital status		
Married	341 (18.4)	131 (16.5)
Single/Domestic Partner	1412 (76.3)	633 (79.8)
DSW	97 (5.2)	29 (3.7)
SES		
Low poverty	744 (40.2)	315 (39.7)
Medium poverty	986 (53.3)	425 (53.6)
High poverty	120 (6.5)	53 (6.7)
YOD		
1990s	325 (17.6)	142 (17.9)
2000s and 2010s	1525 (82.4)	651 (82.1)
EOD		
Confined	56 (3.0)	27 (3.4)
Local Invasion	374 (20.2)	147 (18.5)
Metastasis	179 (9.7)	88 (11.1)
Unknown	1241 (67.1)	531 (67.0)
Site		
Extremity	420 (22.7)	186 (23.5)
Axial Skeleton	718 (38.8)	295 (37.2)
Other	712 (38.5)	312 (39.3)
Tumor Size		
≤ 50 mm	302 (16.3)	138(17.4)
> 50 mm–100 mm	529(28.6)	216 (27.2)
> 100 mm	1019 (55.1)	439 (55.4)
Surgery		
Yes	1126 (60.9)	475 (59.9)
NO/Unknown	724 (39.1)	318 (40.1)
Radiotherapy		
Yes	871 (47.1)	353 (44.5)
No	979 (52.9)	440 (55.5)
Chemotherapy		
Yes	1683 (91.0)	745 (93.9)
NO/Unknown	167 (9.0)	48 (6.1)

*Abbreviations*: *DSW* Divorced, separated and widowed, *YOD* Year of diagnosis, *EOD* Extend of disease, *SES* Socioeconomic Status

### Multivariate cox regression analysis results

Multivariate models were developed to identify independent prognostic variables. Sex, marital status, SES score, YOD, primary site, radiotherapy, and chemotherapy were not associated with the significant differences in survival. Thus, age at diagnosis, race, EOD, tumor size, and surgery were subjected to multivariate Cox regression analysis. The multivariate analysis demonstrated that age at diagnosis > 30 years old (adjusted hazard ratio [12], 2.153; 95% confidence interval [CI], 1.812 to 2.558; *p* < 0.001), being black (aHR, 1.497; 95% CI, 1.054 to 2.128; *p* < 0.05), metastasis (aHR, 4.839; 95% CI, 2.780 to 8.424; *p* < 0.001), unknown EOD (aHR, 2.127; 95% CI, 1.246 to 3.632; *p* < 0.01), tumor size > 50 and ≤ 100 mm (aHR, 1.469; 95% CI, 1.105 to 1.953; *p* < 0.01), tumor size > 100 mm (aHR, 2.273; 95% CI, 1.755 to 2.945; *p* < 0.001), and nonsurgical treatment (aHR, 1.951; 95% CI, 1.670 to 2.280; *p* < 0.001) were independent negative predictors of disease-specific survival (DSS). The multivariate analyses of DSS in the training set are listed in Table 2.

**Table 2:** Multivariate analyses of disease-specific survival in the training set

Variable	Multivariate analysis		
	aHR	95% CI	*p*-value
Age			
≤ 30 years	Reference		
>30 years	2.153	1.812–2.558	0.000***
Race			
White	Reference		
Black	1.497	1.054–2.128	0.024*
Other	1.177	0.894–1.548	0.245
EOD			
Confined	Reference		
Local Invasion	1.692	0.977–2.932	0.061
Metastasis	4.839	2.780–8.424	0.000***
Unknown	2.127	1.246–3.632	0.006**
Tumor Size			
≤ 50 mm	Reference		
> 50 mm–100 mm	1.469	1.105–1.953	0.008**
> 100 mm	2.273	1.755–2.945	0.000***
Surgery			
Yes	Reference		
NO/Unknown	1.951	1.670–2.280	0.000***

*Abbreviations*: *SEER* Surveillance, Epidemiology, and End Result, *aHR* Adjusted hazard ratio, *EOD* Extend of disease

Note: *p < 0.05, ** *p* < 0.01, *** *p* < 0.001

### Nomogram construction

**Fig. 1:**
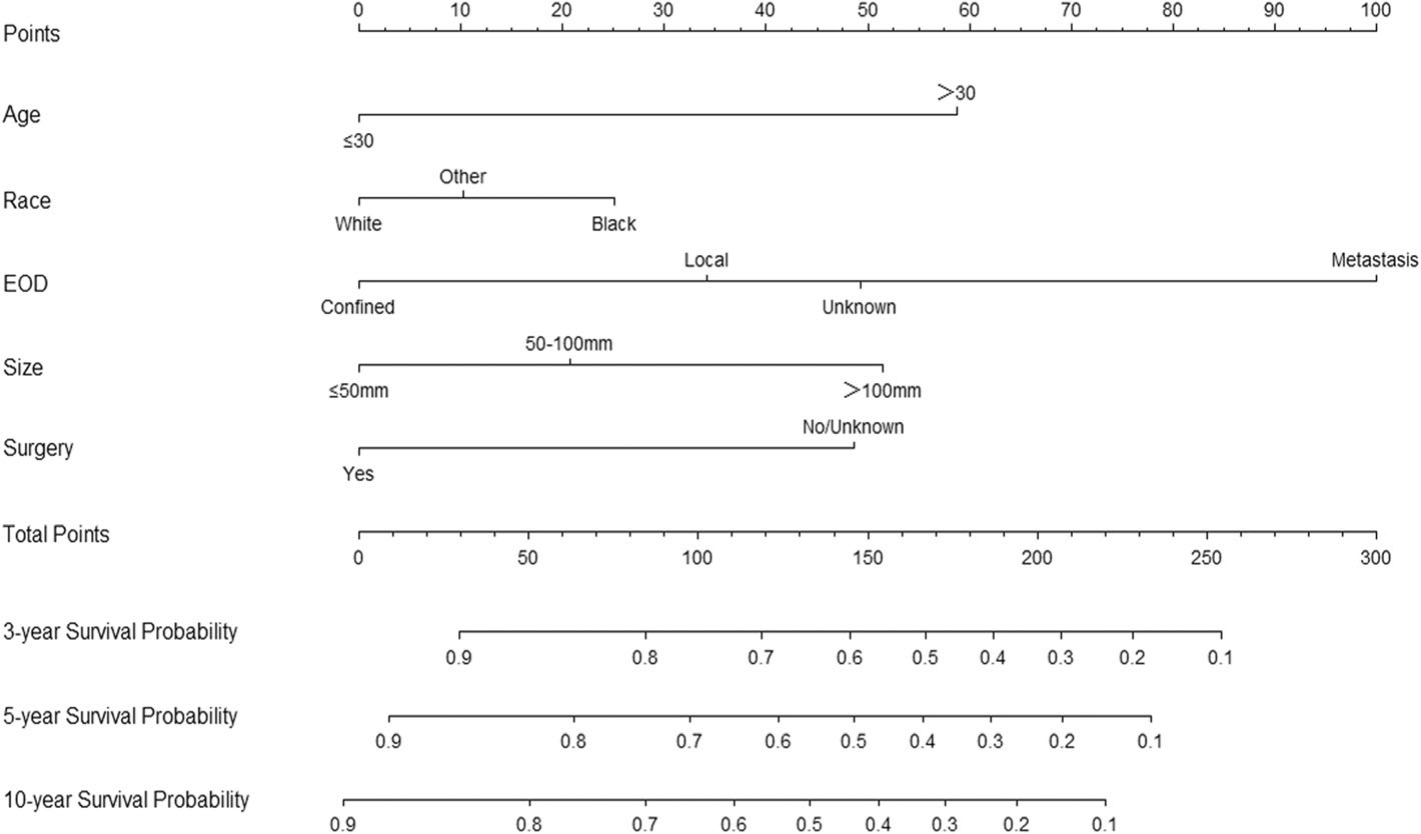
Nomogram predicting 3-, 5-, and 10-year survival. EOD, extent of disease; AS, axial skeleton

The results of the logistic regression model listed in Table 2 were utilized to construct a nomogram (Fig. 1). Each predictor was included in its line according to that scale. The total points on the nomogram were added and then converted into the probability of 3-, 5-, and 10-year survival with guidance from the linear parallel lines. The nomogram showed that metastasis, which had the largest absolute values, was the strongest contributor to the risk of prognosis, followed by age > 30 years old, large tumor (> 100 mm), nonsurgical treatment, local and unknown EOD, being black, intermediate tumor size (> 50 and ≤ 100 mm), and being male. Meanwhile, the protective factors included age ≤ 30 years old, being white, confined tumor, small size tumor (≤50 mm), and surgery performed.

### Performance of the nomogram

**Fig. 2:**
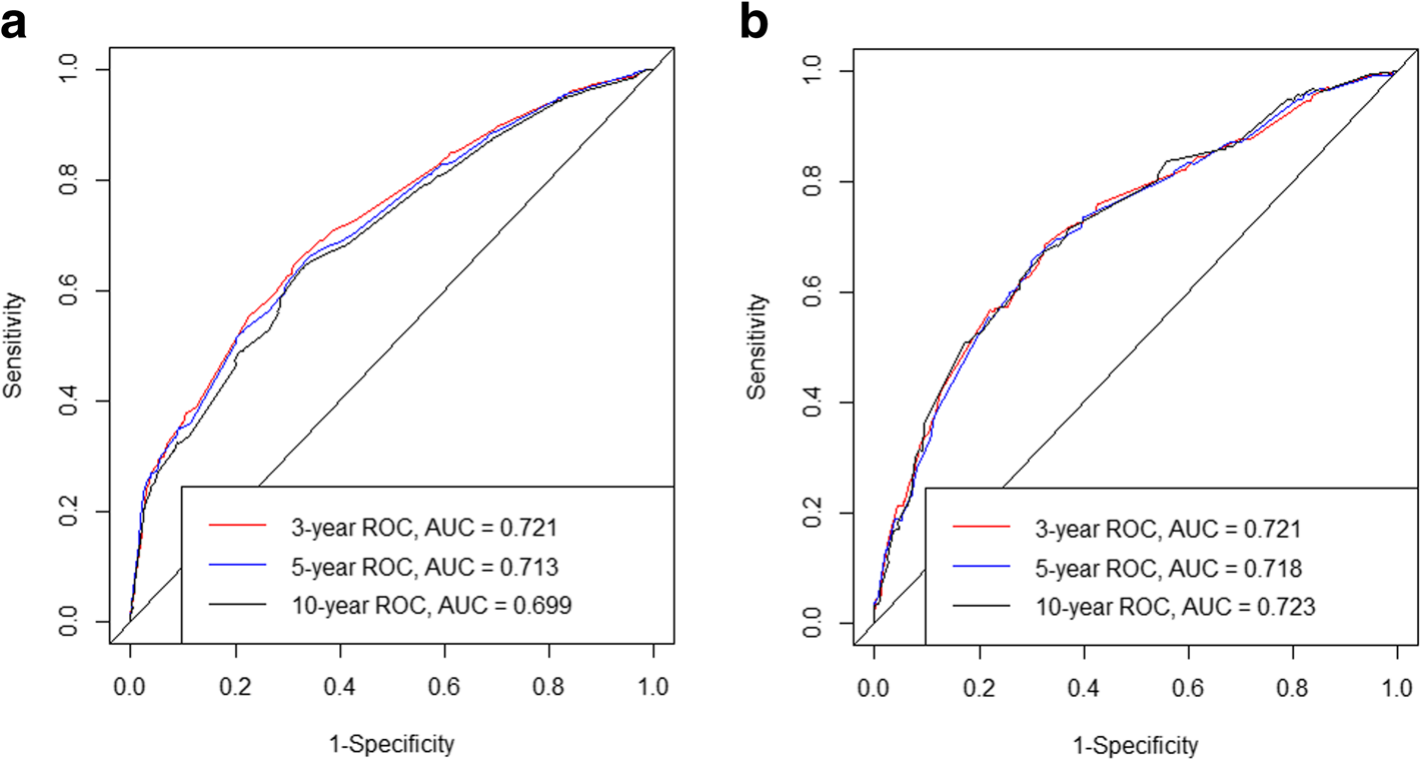
ROC curves. ROC curve analyses were generated to test the performance evaluating of the newly established nomogram, by the areas under the ROC curves (AUC). **a** came from the training set, and **b** came from the validation set

Based on the C-index analysis of the SEER training cohort, the nomogram provided relatively high C-indexes for the 3-, 5-, and 10-year survivals at 0.721, 0.713, and 0.699, respectively; the corresponding values for the external validation cohort were also high at 0.721, 0.718, and 0.723. These findings indicated that the model had good discriminative ability (Fig. 2).

### Validation of the nomogram

**Fig. 3:**
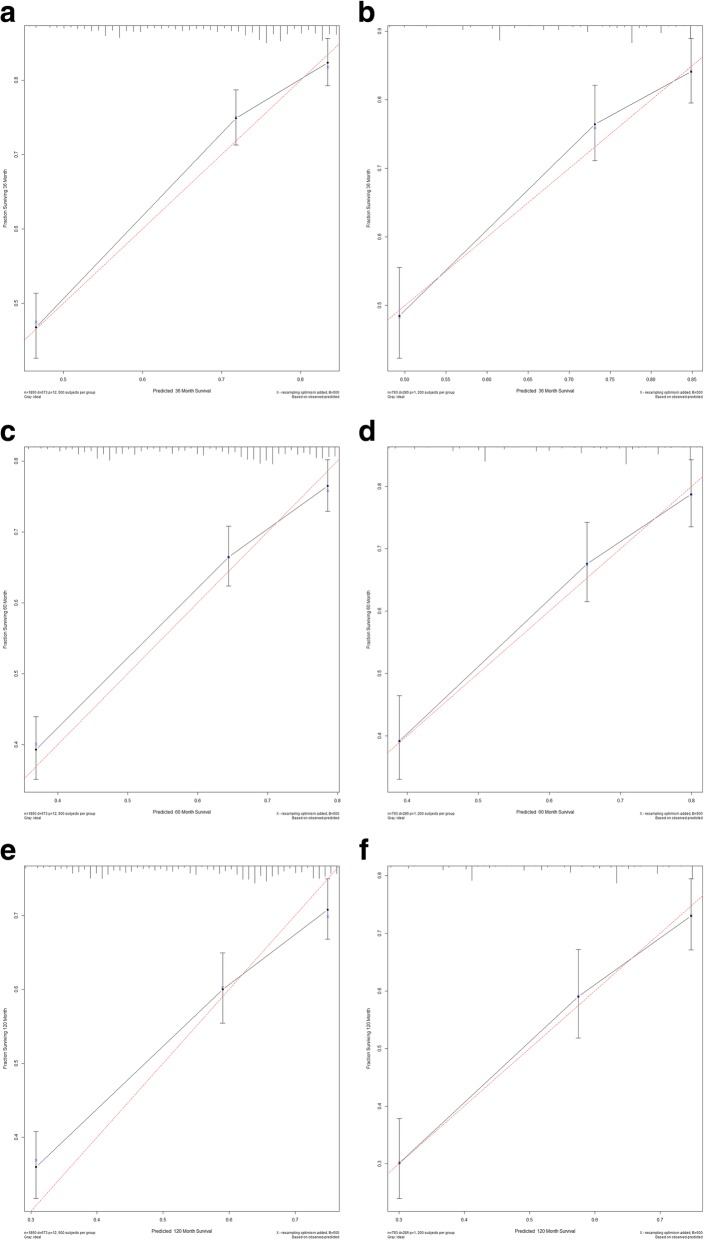
Calibration curves for 3-, 5-, and 10-year survival. Calibration curves depict the calibration of each model in terms of the agreement between the predicted probabilities and observed outcomes of the training set (**a**, **c**, **e**) and validation set (**b**, **d**, **f**)

The new model for the established nomogram included the following variables that were entered into the multivariate Cox regression analysis: age at diagnosis, race, EOD, tumor size, and surgery. The new model that included therapy of surgery provided better C-indexes (0.684 and 0.704 in the training and validation cohorts, respectively) than that of the model without surgery (0.661 and 0.668). A high C-index indicates good ability to separate patients with different survival outcomes. The calibration curves in Fig. 3 depict the calibration of the new model in terms of the agreement between the predicted probabilities and observed outcomes for 3-, 5-, and 10-year survival.

The NRI values were 0.361 (95% CI, 0.241 to 0.525), 0.481 (95% CI, 0.260 to 0.562), and 0.520 (95% CI, 0.350 to 0.569) for 3, 5, and 10 years of follow-up examinations in the training cohort, respectively. In the validation cohort, the NRI values for 3, 5, and 10 years of follow-up were 0.351 (95% CI, 0.242 to 0.502), 0.458 (95% CI, 0.320 to 0.541), and 0.494 (95% CI, 0.350 to 0.670), respectively. These results showed that the new model exhibited superior predictive performance compared with the model without the therapy of surgery. Similarly, the IDI values for 3, 5, and 10 years of follow-up examinations were 0.026, 0.028, and 0.029 in the training cohort and 0.021, 0.023, and 0.025 in the validation cohort, respectively.

### Clinical use

**Fig. 4:**
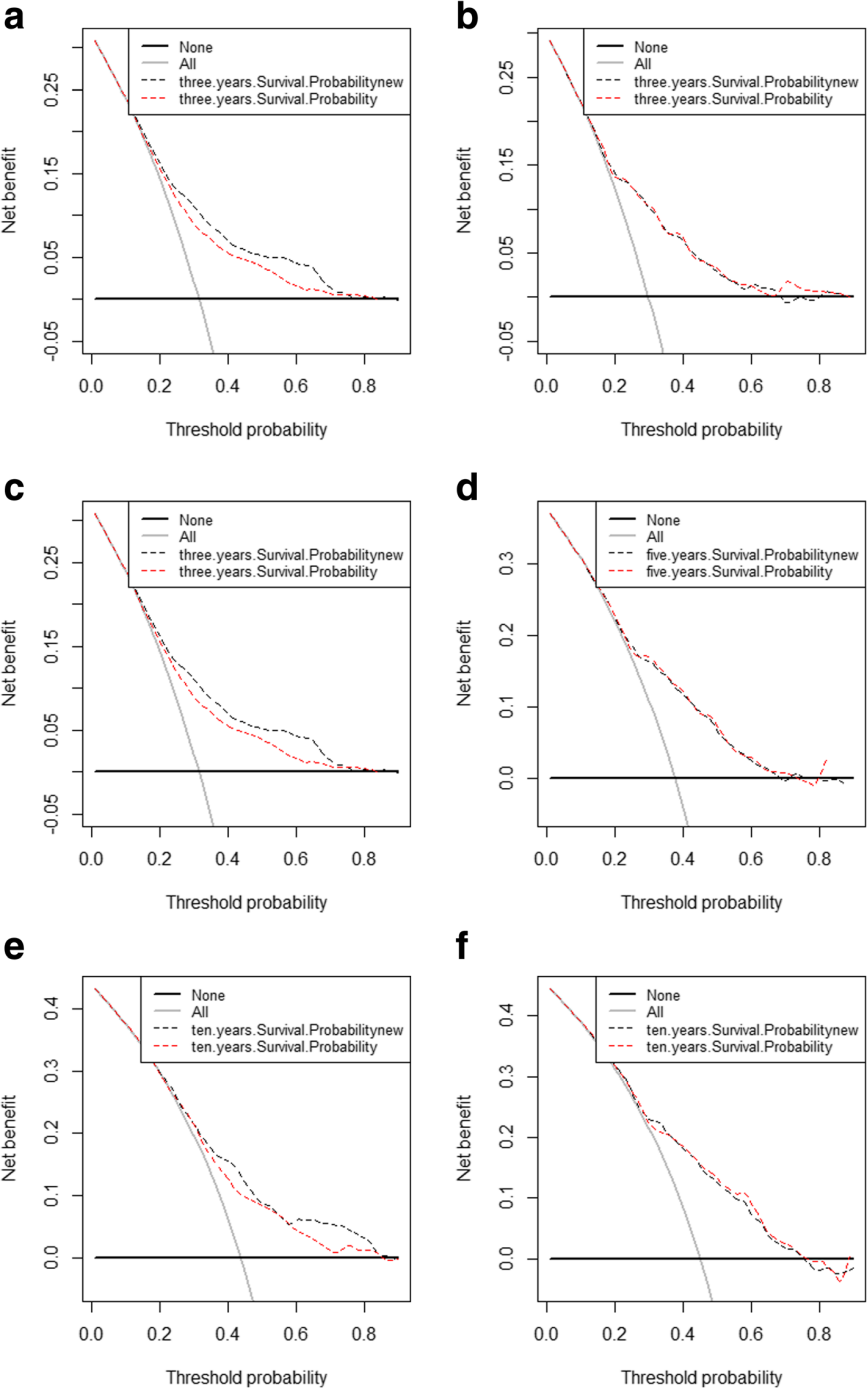
Decision curve analysis of the training set (**a**, **c**, **e**) and validation set (**b**, **d**, **f**) for 3-, 5-, and 10-year survival. In the figure, the blue dotted line represents the DCA of new model, contrastively, the red dotted line represents the DCA of model without therapies. All = Assume all ES patients survive, None = Assume none ES patient survives

DCA graphically showed the large net benefits of the new model for predicting 3-, 5-, and 10-year survival (Fig. 4) to verify its clinical utilization and impact in practical decision-making.

## Discussion

ES is an rare and aggressive type of malignancy that normally develops in young patients from childhood to early adulthood [13]. ES is the second most common primary malignant bone tumor in people younger than 30 years (second only to osteosarcoma) and the most common primary malignant bone tumor in those younger than 10 years. The annual incidence of ES among Caucasians is less than 3 per 1,000,000 [3], thereby indicating that data from single-center studies cannot provide adequate sample sizes. Therefore, this study was based on a large-sample database of the SEER program, which initially started with eight registries in 1973 and has continuously added other participating sites over time. At present, the database includes 18 geographically diverse areas representing 26% of the US population with efforts to reflect the racial, economic, and social diversity of the country as a whole [2, 6, 14]. The neoadjuvant chemoradiation treatment of ES began in the early 1990s [15]. To obtain reliable research results, we identified 2,643 patients with ES in the SEER program database from 1990 to 2015. ES mostly occurs in young people. In our study, most of the patients were ≤ 30 years old, accounting for 78.4 and 79.8% in the training and validation cohorts, respectively. Table 1 presents that most of the patients were male, white, had a marital status of single/domestic partner, diagnosed in the 2000s or 2010s, treated with surgery, and treated with chemotherapy; these results were consistent with previous research findings [16–19]. Although ES has the highest incidence in people under the age of 30 [20], the prognosis is better for those with a younger age of onset and worse for those with a higher age of onset [1]. Similarly, in our nomogram (Fig. 1), the prognosis of people older than 30 was worse than that of people younger than 30. Regarding the cause of this phenomenon, Lee et al. [19] and Grevener et al. [21] found that adult patients received few cases of chemotherapy, and older patients were more likely to have multiple comorbidities, including diabetes, high blood pressure, and secondary cancer, which complicated the situation.

The long-term survival rate of ES for nonmetastatic disease at presentation has improved from 10 to 15% to 60–70% since the early 1990s through the application of multimodality approaches, including surgery, radiotherapy, and neoadjuvant chemotherapy [12, 22, 23]. However, ES exhibits an aggressive behavior that often results in lung metastasis, which is a poor prognostic factor given that only 20% of patients with metastases can survive for a long time [1, 2, 20]. The early identification of high-risk ES patients is helpful in providing adjuvant treatment or trials. Existing clinical staging systems only consider tumor size and histological metastasis. For example, the staging system of the American Joint Committee on Cancer can only estimate the limited clinical risk of ES. Therefore, the use of Cox regression analysis and the developed nomogram provides a comprehensive predictive model that includes not only the system demographics but also the therapy of surgery and other clinical parameters.

A nomogram is a convenient graphical representation of a mathematical model. It provides an intuitive way to combine important factors and predict a specific endpoint. The nomogram is also a reliable tool for quantifying risk and widely used in applied tumor prognoses. A well-developed clinical nomogram is a popular decision-making tool that can be used to predict the outcome of an individual and benefit both clinicians and patients [24]. Nomograms in many studies [2, 16, 17, 20, 25] indicate that being black and aged appear to be high-risk factors. However, small size tumor and surgery treatment demonstrate improved outcomes in DSS for ES. This trend is understandable given the aggressive therapies needed to treat such disease. Patients with metastatic diseases at the initial presentation have worse prognoses than those with confined diseases [2, 13, 18, 25]. Knowledge of these features will be helpful in clinical decisions.

Similar to previous studies [10, 26, 27], we applied IDI and NRI to evaluate whether the newly constructed prognostic model performed well and whether it should be used in clinical practice. Compared with radiotherapy and chemotherapy, surgery is the most effective means of treating ES [2, 18]. The new model containing the therapy of surgery showed good discrimination and calibration, in which both IDI and NRI for 3, 5, and 10 years of follow-up examinations showed improved C-index, as mentioned in the Results section.

Finally, our newly constructed nomogram model included a wide range of clinical risk factors, namely, age at diagnosis, race, EOD, tumor size, and surgery, which were easily available and routinely collected from historical records. Figure 4 shows the results of our DCA, wherein the abscissa and ordinate are the threshold probability and net benefit rate, respectively [28–31]. To the best of our knowledge, this study is the first to use IDI, NRI, and DCA in the verification of the predictive abilities of nomograms for ES. Thus, the nomogram is helpful to accurately predict the 3-, 5-, and 10-year survivals of ES patients.

### Limitations

First, important prognostic factors, such as tumor markers and the expression of the TP53 gene, were not available in the SEER database. Second, information was not available for some of the cases. Hence, we could only define the subclassifications as unknown, such as for EOD and tumor size. Third, similar to other malignant bone tumors, ES showed unavailable AJCC/TNM data in the SEER database that might have affected the diagnostic and predictive accuracy of our new tool [32]. Finally, rather than representing absolutely accurate prognoses, the predicted values calculated from the nomogram were only suitable for interpretation by clinicians. Future studies can use the present findings to develop a well-accepted risk prediction tool for ES [33].

## Conclusions

Nomograms are an important component of modern medical decision-making. We developed a reliable nomogram for determining the prognosis and treatment outcomes of ES patients in the US. However, external data verification is still required in future applications, especially for regions outside the US.

## Data Availability

Limited Use Agreement for Surveillance, Epidemiology, and End Results (SEER) Program (https://seer.cancer.gov) SEER*Stat Database: released in April 2017, based on the November 2016 submission. The data can be used publicly.

## Abbreviations

AUC: Area under the curve
DCA: Decision curve analysis
DSW: Divorced, separated and widowed
ES: Ewing sarcoma
IDI: Integrated discrimination improvement
NRI: Net reclassification improvement
OS: Overall survival
SEER: Surveillance, Epidemiology, and End Results
SES: Socioeconomic status
YOD: Year of diagnosis
